# Evaluation of Surface Impact Properties of Thermoplastics: Mechanical Correlation Between Critical Expansion Stress and Uniaxial Tensile Strength

**DOI:** 10.3390/polym18131658

**Published:** 2026-07-03

**Authors:** Tetsuo Takayama, Koki Tsuchiya, Akito Endo

**Affiliations:** 1 Graduate School of Organic Materials Science, Yamagata University, Yamagata 992-8510, Japan; 2 Faculty of Engineering, Yamagata University, Yamagata 992-8510, Japan

**Keywords:** critical expansion stress, DuPont impact test, hydrostatic stress, staircase method, void formation

## Abstract

For the impact-resistance evaluation of thermoplastics, the DuPont impact test is widely used to replicate multiaxial stress states inherent in actual product environments. However, conventional evaluation methods remain constrained by probabilistic pass/fail judgments or empirical calculations of absorbed energy. Consequently, quantifying the “material-specific fracture criterion,” which is indispensable for high-fidelity computer-aided engineering (CAE) analysis, persists as an important challenge. While our previous works established the derivation of CES from uniaxial tensile tests, the core originality of this study lies in extending this mechanical framework to the dynamic and multiaxial stress states of the DuPont impact test. By integrating a mathematical model with the probabilistic results of the staircase method, we enable for the first time the quantitative identification of material-specific fracture thresholds directly from standard drop-weight impact configurations. For this study, a novel mechanical model for deformation and fracture behavior in the DuPont impact test is constructed. Then a quantitative evaluation method is proposed for the “Critical Expansion Stress (CES),” a material-specific threshold triggering fracture under multiaxial stress. Specifically, using thermoplastic materials of five types and seven grades (including PP, POM, PS, ABS, and PC), the surface impact energy absorbed per unit volume was calculated via the DuPont impact test using the staircase method, accounting for size effects. Furthermore, microscopic parameters (shear modulus G and critical void volume fraction f0) were identified theoretically based on the mechanical properties obtained from short-beam shear tests. These parameters were integrated into a mathematical model to derive the CES. Comparing the derived CES with the true-stress-based uniaxial tensile strength, which incorporates the necking behavior during large deformations, revealed a distinct correlation governed by their mechanical relation (the 1:3 rule) based on the theoretical definition of hydrostatic stress. For the highly ductile polymer exhibiting significant strain hardening, this correlation holds universally when evaluated at the initial plastic flow stage prior to massive molecular orientation. The proposed method serves as a practical quantitative screening tool for evaluating the surface impact characteristics of plastic materials, providing an accessible framework for identifying material-specific fracture thresholds.

## 1. Introduction

Thermoplastics, because of their excellent balance of lightweight properties, moldability, and mechanical performance, are applied widely to structural components requiring high impact resistance, such as automotive exterior and interior parts, and to various safety equipment [1]. For these product designs, predicting fracture behavior against sudden external loads such as collisions or drops and ensuring product reliability are of paramount importance. Conventionally, the notched Charpy impact test and the notched Izod impact test have been employed widely to evaluate the impact resistance of plastic materials [2,3]. However, these testing methods evaluate crack propagation behavior under one-dimensional stress concentration at the tip of an artificially introduced notch. Consequently, they fail to represent the deformation and fracture processes accurately under the “planar impact loading (multiaxial stress states)” which actual products experience.

As a methodology for evaluating impact resistance under multiaxial stress states that closely resemble actual operating environments, drop-weight impact tests, typified by the DuPont impact test, have been used widely. However, conventional DuPont impact testing remains confined to empirical and qualitative evaluations, such as probabilistic pass/fail judgments or the calculation of macroscopic fracture potential energy, which depends heavily on the specimen thickness and apparatus geometry [4]. In modern product design, which demands advanced impact analysis using computer-aided engineering (CAE), a robust mechanical model capable of directly deriving and identifying testing-condition-independent “material-specific fracture criteria” from drop-weight impact tests involving multiaxial and dynamic deformation has yet to be established. Consequently, the inherent scatter in test results and the ambiguity of macroscopic evaluation criteria have posed daunting practical barriers to their application in high-fidelity CAE analysis [4].

To predict such failure behavior under complex stress fields mathematically, outstanding pioneering studies such as the craze initiation theory by Bucknall et al. and the multiaxial failure criteria by Altenbach et al. have pushed efforts forward [5,6]. Nevertheless, these classical failure criteria require sophisticated stress control and vast amounts of characterization by experimentation, posing severe engineering challenges for practical implementation. While these classical failure criteria provide a rigorous theoretical basis, their primary inadequacy lies in the requirement for specialized testing equipment capable of sophisticated multi-axial stress control and the need for extensive parameter fitting across numerous experimental conditions. These requirements pose significant practical barriers for rapid material screening. In contrast, the measurable advantage of our proposed framework is its ability to derive a robust multiaxial fracture criterion (CES) using only a few parameters obtained from straightforward, standard mechanical tests, such as the short-beam shear test. This approach bridges the gap between complex damage mechanics and practical industrial application.

To break through these practical limitations, this study uses microscopic mechanical parameters obtained from a straightforward short-beam shear test, thereby achieving an exceptionally simple yet robust quantitative formulation of the multiaxial fracture criterion (Critical Expansion Stress). By balancing outstanding ease of measurement with high compatibility with numerical simulations, this framework bridges the gap separating conventional macroscopic tests and complex theoretical criteria.

The macroscopic yield and fracture mechanisms of polymer materials are predominantly governed by two competing elementary processes: “shear yielding,” which follows the von Mises yield criterion, and “craze and void formation,” which involves volume expansion induced by hydrostatic stress (multiaxial tensile stress) [7,8]. From earlier studies, the authors developed a “theory of shear yield initiation stress” that accounts for polymer-specific molecular friction, thereby elucidating the theoretical framework of mechanical responses under shear deformation [9]. Furthermore, to comprehend macroscopic fracture phenomena, it is indispensable to quantify the microscopic initial defects and structural characteristics within the material. To address this issue, the authors have proposed a “mechanical property evaluation method using short-beam shear tests” and have already established a technique for accurate isolation and extraction of the void fraction f_0_ and shear modulus G, which reflect the microscopic structural characteristics of the material, from a unique stress field [10]. These preceding findings suggest that the mechanical properties of plastic materials exhibiting complex deformation behavior can be described based on microscopic parameters.

The impact strength of thermoplastics is classified broadly into two categories, each characterized by distinct fracture mechanisms: “planar impact” and “notched impact.” As a method for evaluating the planar impact strength of plastic plates and film-like molded products, the DuPont drop-weight impact test has been adopted widely [11]. This testing method is a straightforward approach: a weight is dropped via an indenter to judge the presence or absence of specimen failure. The method provides the benefit of replicating loading modes that closely resemble actual product operating environments. However, it is inherently a probabilistic test based on a binary pass/fail judgment. Consequently, it entails a fundamental shortcoming: unlike Izod or Charpy impact tests, it cannot directly measure fracture energy or impact strength as a physical quantity from individual specimens. Conventionally, the “50% failure height” or “50% failure energy” calculated as the DuPont impact value serves merely as a relative index under specific testing conditions. The inability to evaluate this index quantitatively as a material-specific fracture toughness value has persisted as a major barrier to materials design.

To process the scatter in DuPont impact test results statistically and to calculate the 50% failure energy, the “staircase method” (or up-and-down method), which is recommended by JIS and ASTM standards, has been used widely [11]. This excellent statistical approach is capable of efficiently identifying the 50% failure threshold by increasing or decreasing the impact energy (the drop height of the weight) applied to the subsequent specimen by a constant step size, depending on the success or failure of the immediately preceding specimen. Pollak and Smith [12], Alsadon et al. [13], Niu et al. [14], and Meeker et al. [15] have reported that thresholds at a 50% failure probability can be derived reliably from both destructive and non-destructive test results. However, the values obtained using the staircase method remain structure-sensitive macroscopic indicators that depend heavily on the testing system geometry, such as specimen thickness, indenter tip radius, and die-hole diameter. Consequently, they do not directly represent the bulk mechanical properties inherent to the material itself. For instance, even for the exact same material, the 50% failure energy can differ considerably for various specimen thicknesses. Consequently, direct input of the obtained data as material parameters (material-specific thresholds) into numerical simulations such as CAE has been impossible.

Under planar impact conditions such as the DuPont impact test, a biaxial tensile stress field is generated within the plane, inducing a high hydrostatic stress component because of constraints in the thickness direction [2]. In such a multiaxial stress field, brittle fracture triggered by void formation (expansion fracture) tends to be more dominant than shear yielding [8,16]. Therefore, for fundamental understanding of planar impact characteristics, it is crucially important not only to measure macroscopic absorbed energy but also to evaluate the “Critical Expansion Stress (CES)” quantitatively, which is a material-specific threshold acting as a microscopic trigger for fracture. The authors earlier proposed an “evaluation method for critical expansion stress via uniaxial tensile testing.” After establishing a procedure to derive the critical expansion stress from the relation between true stress and expansion strain under uniaxial stress, they reported its validity [17]. However, the DuPont impact test involves high strain rates and complex multiaxial stress states: a theoretical model that integrates macroscopic impact test results with microscopic mechanical parameters to derive the critical expansion stress analytically remains unexplored territory.

Based on the previously described academic background and the authors’ earlier findings, this study was conducted to construct a novel mechanical model for the deformation and fracture behavior in DuPont impact testing, thereby establishing a valid quantitative evaluation method for the CES under multiaxial impact loading. By fusing statistical processing via the staircase method with a fracture mechanics model under biaxial extension, this proposed methodology estimates the material-specific critical expansion stress from DuPont impact test results, which have conventionally been considered incapable of supporting quantitative evaluation.

Specifically, for a total of five types and seven grades of thermoplastic materials (including PP, PC, POM, PS and ABS), the surface impact energy absorbed per unit volume is calculated via the DuPont impact test. Simultaneously, microscopic parameters (shear modulus G and critical void volume fraction f_0_) for each material are acquired using a previously reported short-beam shear test. These physical quantities are then integrated into a newly proposed theoretical equation to derive the critical expansion stress. Finally, the critical expansion stress calculated under dynamic impact is compared with the tensile strength evaluated from uniaxial tensile tests. By demonstrating that their mechanical relation governed by the theoretical definition of hydrostatic stress (the “1:3 rule”) holds true, we verify that the proposed approach serves as a practical quantitative evaluation guideline for the surface impact characteristics of plastic materials. In summary, although previous studies have successfully extracted microscopic parameters (G and f_0_) using short-beam shear tests and evaluated CES under quasi-static uniaxial tension, a critical gap remains: the lack of a quantitative methodology to derive these ‘material-specific criteria’ from complex, high-speed planar impact tests. This study fills this gap by fusing statistical staircase analysis with a multi-axial fracture mechanics model, providing a robust tool for high-fidelity CAE analysis that transcends the qualitative limitations of conventional DuPont testing.

## 2. Materials and Methods

### 2.1. Materials

For this study, five types and seven grades of resins with markedly different inherent deformation/fracture modes (brittle/ductile behaviors) and fracture susceptibilities were selected as the evaluation targets to verify the universality of the fracture initiation mechanism under multiaxial stress states in thermoplastic polymers. This material group is mapped out systematically to clarify not only differences in the matrix aggregation states (crystalline/amorphous) but also the competing processes between materials exhibiting a “craze-type (volume expansion-dominant)” fracture mechanism, where void initiation and growth proceed under hydrostatic tensile stress, and materials exhibiting a “shear yielding-type (shape change-dominant)” fracture mechanism, for which microscopic plastic flow is most important.

Polypropylene (PP) and polyoxymethylene (POM) were employed as crystalline polymers that serve as shear yielding-type materials. Their void expansion is suppressed by the strong crystalline constraints of the matrix, yet their shear yield characteristics change drastically under high-rate deformation. For PP, two types were adopted: Novatec PP (N-PP, grade MA1B; Japan Polychem Corp., Tokyo, Japan); and Novatec MA3H (HM-PP), which was highly rigidified using nucleating agents. For POM, Tenac 3010 (Asahi Kasei Corp., Tokyo, Japan) was used.

Regarding amorphous polymers, polystyrene (PS, Toyo Styrene G210C; Toyo Styrene Co., Ltd., Chiba, Japan) was used as a typical craze-type material that initiates easily and grows crazes (aggregates of microscopic microvoids) under low stress, leading to fracture without macroscopic plastic deformation of the entire matrix. In contrast, two grades of polycarbonate (PC, Iupilon; Mitsubishi Engineering-Plastics Corp., Tokyo, Japan) were supplied as representative ductile (shear yielding-type) materials in which crazing (fracture and coalescence of void fibrils) is suppressed effectively by a high entanglement network density, thereby exhibiting extensive shear yielding. Here, the high-molecular-weight (high-viscosity) grade S2000 was designated as HV-PC. The low-molecular-weight (low-viscosity) grade H3000 was designated as LV-PC. They were used to evaluate the effects of differences in entanglement density on the competition between fracture mechanisms. Furthermore, an acrylonitrile–butadiene–styrene copolymer (ABS, Kralastic GA-101; Nippon A&L Inc., Osaka, Japan) was used as a rubber-modified material with a hybrid fracture susceptibility. In this material, cavitation (voiding) of rubber particles occurs preferentially under low stress, but subsequent void expansion is constrained by local shear yielding of the surrounding matrix.

By covering these diverse thermoplastic polymers comprehensively with different fracture initiation triggers and deformation modes, it became possible to verify how universally the continuum mechanics-based “1:3 rule” (the mechanical correlation between uniaxial tensile strength and critical expansion stress) holds true, transcending differences in the materials’ microscopic structures and fracture susceptibilities. Details of each material used for this study are presented in Table 1.

### 2.2. Injection Molding

These materials were subjected to injection molding after being pre-dried according to the recommended conditions for each resin. Specimen preparation was performed using an ultra-compact electric injection molding machine (C, Mobile0813; Shinko Sellbic Co., Ltd., Tokyo, Japan). By changing the molds, specimen shapes of two types were molded according to the intended tests. For the impact and shear tests, disc-shaped specimens with 30 mm diameter and 2 mm thickness were prepared. For the tensile test, by contrast, dumbbell-shaped specimens were prepared as recommended by ISO 527, corresponding to a 1/4 scale of the multipurpose dumbbell specimen [18]. The injection molding conditions for each resin were selected to yield defect-free products consistently without appearance defects such as sink marks, voids, or short shots. Table 2 presents the specific molding conditions: pre-drying conditions, cylinder temperature, mold temperature, injection speed, holding pressure, etc.

### 2.3. DuPont Impact Tests

To evaluate the impact resistance of the resins, drop-weight impact tests were performed using a DuPont impact tester (Myz Tester Co., Ltd., Kyoto, Japan). For this test, the previously described disc-shaped specimen was placed on an anvil with a radius of 6.35 mm. Then an impact load was applied to the specimen by allowing a weight to fall freely through a 1/2-inch-diameter impactor (1.3 cm). Three weight masses (m) were used: 300 g, 500 g, and 1000 g. The drop height (h) was adjustable from 50 mm to 500 mm in 50 mm increments. The selection of weight masses (300 g, 500 g, and 1000 g) and drop heights (50 mm to 500 mm) was determined through preliminary trials to ensure that a stable and valid staircase sequence could be established for all seven polymer grades. The evaluation was conducted using the staircase method (up-and-down method). The 50% impact failure energy (E_50_), at which the specimen fractures with a 50% probability, was calculated from results of at least 20 tests. The impact energy (E) is expressed by the following Equation (1) using the mass of the weight (m), gravitational acceleration (g), and the drop height (h).

(1) E=mgh

For this study, the 50% impact failure energy density (E_50_) and its standard deviation were calculated based on the statistical method proposed by Dixon and Mood (Dixon–Mood method) [19]. Details of the analysis are presented hereinafter.

From the test results, the event with the lower frequency of occurrence (either “failure” or “non-failure”) is selected as the analysis target. The test levels at which the target event was observed are indexed in ascending order as i = 0, 1, 2,…k. The number of occurrences of the event at each level is signified by n_i_.

The total number of events used for the analysis (N) and the statistical quantities (X and Y) are defined by the following Equations (2)–(4).

(2) $$N=\sum_{i=0}^{k}n_{i}$$

(3) $$X=\sum_{i=0}^{k}i\cdot n_{i}$$

(4) $$Y=\sum_{i=0}^{k}i^{2}\cdot n_{i}$$

Therein, k is the index of the highest level at which the event was observed.

The 50% impact failure energy obtained from the staircase method, initially calculated in Joules [J] via Equation (1), was converted into the surface impact energy absorbed per unit volume (E_50_, [MJ/m^3^]) to eliminate the geometric size effects of the specimens and the testing apparatus. The failure energy density E_50_ is defined as

(5) $$E_{50}=\frac{E_{failure}}{V_{effective}}=\frac{X_{0}+d\left(\frac{X}{N}\pm \frac{1}{2}\right)}{AB},$$

where E_failure_ stands for the total 50% failure energy [J], and V_effective_ represents the effective deformation volume [m^3^] defined as V_effective_ = A·B. Here, A represents the projected area of the indenter tip (πr^2^, where r = 6.35 mm for the 1/2-inch diameter indenter), and B represents the specimen thickness (2.0 mm). This effective volume (A·B) was selected based on the assumption that the impact energy is dissipated predominantly through compressive and shear deformations directly beneath the localized contact zone of the indenter during the initial stage of planar impact, before the catastrophic radial crack propagation. Also, X_0_ is the physical quantity (such as energy) of the lowest level at which the target event was observed; d is the step size between test levels (the increment/decrement interval between levels). Regarding the ± sign in the equation, + is chosen when the target event is “non-failure”; − is chosen when it is “failure”.

Furthermore, the standard deviation (s), which represents the variance of the test results, was estimated using the following equation:

(6) $$s=\frac{1.620\cdot d\left(\frac{NY\;-\;X^{2}}{X^{2}}+0.029\right)}{AB}$$

This test was repeated at least 20 times. It was continued until both events occurred 10 times or more. The event with the lower number of occurrences in the test was selected as the target event for analysis.

It is noteworthy that calculation of the analytical failure thresholds in the subsequent sections involves coupling of the dynamic failure energy obtained here (E_50_) with micro-mechanical parameters derived from quasi-static shear and tensile tests. Based on our scaling framework outlined in Section 1, the rate-dependent variations in both the dynamic energy response and the matrix rigidity are assumed to compensate one another during the ratio calculation process. This assumption allows the seamless integration of quasi-static material baselines into the dynamic impact formulation, provided that the reference stresses reflect the equivalent deformation stage before massive strain hardening occurs.

It should be noted that while the actual drop-weight impact process involves complex stress components including local compression, bending, and contact stresses, this study specifically focuses on the ‘expansion fracture’ triggered by high hydrostatic stress components arising from thickness-direction constraints. Under this assumption, we designate the Critical Expansion Stress (CES) as the dominant governing factor for the initiation of macroscopic failure in these thermoplastic plates.

### 2.4. Short-Beam Shear Tests

**Figure 1:**
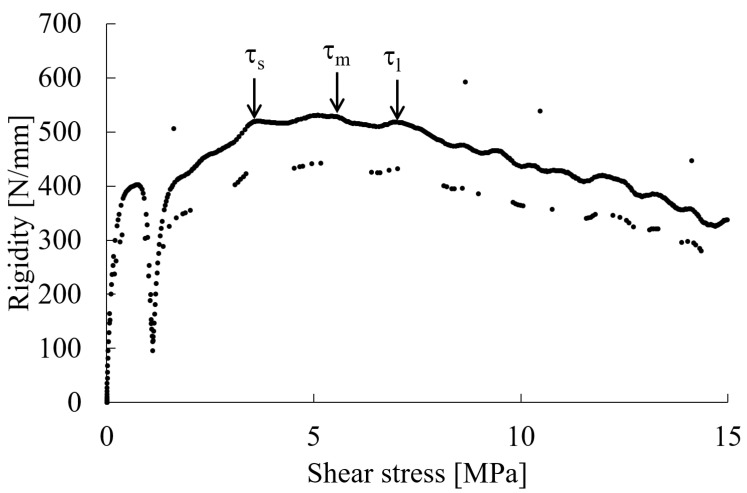
Explanatory diagram of the method for determining the characteristic stresses (τ_s_, τ_m_, τ_l_) on the rigidity—shear stress curves.

To evaluate the internal shear properties of the molded products, short-beam shear tests were performed using a desktop tensile and compression testing machine (MCT-2150; A&D Co., Ltd., Tokyo, Japan). The specimens were 10-mm-wide strip-shaped pieces machined from the central portion of the disc-shaped specimens. The test was conducted in a three-point bending configuration with the span length set as 10 mm. The crosshead speed was set as 10 mm/min. The span length of 10 mm and the crosshead speed of 10 mm/min were chosen based on the authors’ previously validated methodology [10]. These parameters are specifically designed to generate the unique internal shear stress field required for the accurate extraction of the microscopic parameters (G and f_0_) from disc-shaped injection-molded specimens. The load was applied using the gate portion of the specimen as the loading zone (indenter contact area). Stiffness was found by differentiating the load–displacement curve obtained from this test with respect to displacement. A stiffness–load curve was constructed, an example of which is portrayed in Figure 1. From this curve, three points at which the stiffness discontinuously decreased were extracted, excluding the first maximum point. Using the loads at these points, the shear stress was calculated according to the following Equation (7).

(7) $$\tau =\frac{3}{4}\frac{P}{BW}$$

Here, P, B, and W respectively signify the load, the specimen thickness, and the specimen width. As reported earlier by the authors, the composite component of the shear stress at each point represents the shear stress at the onset of yielding, which can be found using Equation (8) [20].

(8) $$\tau_{y}=\sqrt{\tau_{s}^{2}+\tau_{m}^{2}+\tau_{l}^{2}}$$

Furthermore, they reported that the Poisson’s ratio (υ) is obtainable using the following Equation (9) [21].

(9) $$\upsilon =\frac{\tau_{s}+\tau_{m}}{4\tau_{l}}$$

The authors reported earlier that the shear stress at the onset of yielding is explainable using the following Equation (10) [7].

(10) $$\tau_{y}=\left(T_{inj}-T_{test}\right)\cos\left(\tan^{-1}2\upsilon \right)\sqrt{\frac{15E\left(1-2\upsilon \right)\left(1+\upsilon \right)}{\left(1-\upsilon \right)}}$$

In that equation, T_test_ and T_inj_ respectively represent the test temperature (23 °C) and the injection molding temperature. Solving this Equation (10) for Young’s modulus yields the following Equation (11).

(11) $$E={\left(\frac{\tau_{y}}{\left(T_{inj}-T_{test}\right)\cos\left(\tan^{-1}2\upsilon \right)}\right)}^{2}\frac{\left(1-\upsilon \right)}{15\left(1-2\upsilon \right)\left(1+\upsilon \right)}$$

Given that the core layer of injection-molded products possesses mechanical properties close to isotropy, the shear modulus G can be found using the following Equation (12).

(12) $$G=\frac{E}{2\left(1+\upsilon \right)}$$

Through the procedure described above, Young’s modulus, Poisson’s ratio, the shear stress at the onset of yielding, and the shear modulus were calculated as evaluation parameters. The short-beam shear test was performed at least five times for each sample. Then the average value and standard deviation for each parameter were determined.

### 2.5. Universal Tensile Tests

To evaluate the tensile properties, tensile tests were performed using a compact universal mechanical testing machine (FSA-1KE-1000N-L; Imada Co., Ltd., Aichi, Japan). Dumbbell-shaped specimens were used for the tests. The gripping distance (gauge length) was set as 22 mm. The crosshead speed was 150 mm/min, which corresponds to a strain rate of 2.04/min (0.034/s) based on the initial gripping distance. Based on the results of the preliminary test, this crosshead speed was selected because it is the maximum rate that can be reliably measured using the testing machine. Tensile Modulus (TM) and Poisson’s ratio in tension (υ_t_) were evaluated by determining the nominal stress and logarithmic strain using the load–displacement data obtained from this test [22]. Furthermore, for accurate evaluation of the large deformation behavior of the materials, the tensile strength was evaluated after converting it into true stress. Generally, the relational equation for obtaining the true stress (σ_t_) from the nominal stress (σ_n_) and the logarithmic strain (ε_t_) is the following Equation (13).

(13) $$\sigma_{t}=\sigma_{n}\frac{e^{\varepsilon_{t}}}{1+\varepsilon_{v}}$$

Here, ε_v_ is the volumetric strain generated during the tensile test, which was determined by the following Equation (14).

(14) $$\varepsilon_{v}=e^{\varepsilon_{t}\left(1-2\upsilon_{t}\right)}-1$$

Therein, υ_t_ is the Poisson’s ratio obtained from tensile test results.

Standard evaluations conforming to conventional regulations recommend determining the nominal stress. However, this study proposes the following model so that the true stress is obtainable even when neck-in or strain hardening occurs.

First, when neck-in occurs, a local cross-sectional change takes place. However, it is assumed that the true stress does not increase during this process. By introducing this assumption, the cross-sectional area after neck-in can be found using the following Equation (15).

(15) $$A^{\prime }=\frac{\sigma_{to}}{P^{\prime }}\frac{1+\varepsilon_{v}^{\prime }}{e^{\varepsilon_{t}^{\prime }}}$$

In that equation, σ_to_ and P’ respectively represent the true stress and load at the onset of necking. The prime (′) denotes the point at which necking ends. The nominal stress after neck-in is obtainable by recalculating the nominal stress with the cross-sectional area changed from A to A′. As described in this paper, the true stress after neck-in was determined using this recalculated nominal stress.

By ensuring these procedures, we considered that the tensile strength could be determined in terms of true stress from the tensile test results. The tensile test was performed at least five times for each sample. The average value and standard deviation for each parameter were determined.

## 3. Results

### 3.1. Impact Failure Energy Density Determined Using the Staircase Method

DuPont impact tests were conducted for comprehensive evaluation and comparison of the impact-resistance properties of the various selected polymer materials. The evaluated materials included N-PP, HM-PP, POM, PS, ABS, LV-PC, and HV-PC. Table 3 presents the obtained impact failure energy density values, along with their standard deviations (s). The measurements revealed distinct variations in impact failure energy density among the different material codes.

The maximum impact failure energy density was found for the polypropylene materials: N-PP exhibited a value of 0.229 ± 0.016 MJ/m^3^; HM-PP demonstrated a nearly comparable value of 0.227 ± 0.004 MJ/m^3^. Following these, POM showed the third highest impact failure energy density at 0.203 ± 0.043 MJ/m^3^, while simultaneously exhibiting the largest standard deviation among all specimens, indicating wide scattering of the data. In contrast, the impact failure energy density of PS was the lowest at 0.042 ± 0.004 MJ/m^3^, which corresponds to approximately 18% of the value recorded for N-PP. ABS was found to have an intermediate value of 0.090 ± 0.012 MJ/m^3^.

Furthermore, wide differences were found among the polycarbonate specimens, depending on the material formulation. Specifically, whereas HV-PC showed an impact failure energy density of 0.172 ± 0.023 MJ/m^3^, LV-PC exhibited a markedly lower value of 0.056 ± 0.003 MJ/m^3^, which indicates that the impact failure energy density of HV-PC was approximately 3.1 times higher than that of LV-PC under the present test conditions. Overall, the measured values spanned a wide range from a minimum of 0.042 MJ/m^3^ (PS) to a maximum of 0.229 MJ/m^3^ (N-PP), highlighting considerably large material-dependent differences in physical properties.

### 3.2. Mechanical Properties Against Shear Obtained from Short-Beam Shear Tests

To evaluate the microscopic deformation behavior and the anisotropy of mechanical properties in various polymer materials, the yield onset shear stresses in each direction (τ_s_, τ_m_, τ_l_), the shear yield stress (τ_y_), Poisson’s ratio (υ), Young’s modulus (E), and the shear modulus (G) were calculated using short-beam shear tests and other evaluations. Table 4 shows the yield onset shear stress, Young’s modulus, Poisson’s ratio, and shear modulus obtained from the short-beam shear test results.

Remarkable differences in elastic modulus and yield stress were observed among the various polymer materials. Among all the samples, low-viscosity polycarbonate (LV-PC) exhibited the highest Young’s modulus (E = 954 MPa) and shear yield stress (τ_y_ = 19.1 MPa). Polystyrene (PS) also demonstrated comparably high stiffness (E = 941 MPa) and high shear stress behavior. However, non-modified polypropylene (N-PP), a commodity plastic, recorded the lowest Young’s modulus (E = 485 MPa) and shear yield stress (τ_y_ = 12.3 MPa). From comparison of N-PP with high-rigidity polypropylene (HM-PP), HM-PP showed higher values across all shear stresses and elastic moduli, with higher E (638 MPa vs. 485 MPa) and higher τ_y_ (13.4 MPa vs. 12.3 MPa). Regarding polycarbonate (PC), it was confirmed that the low-viscosity grade (LV-PC) possessed higher stiffness and shear yield strength than the high-viscosity grade (HV-PC). The ABS resin exhibited a lower Young’s modulus (E = 512 MPa) and lower shear yield stress (τ_y_ = 12.7 MPa) compared to engineering plastics such as POM and PC. Poisson’s ratio (υ) was measured as falling within the range of 0.324 (N-PP) to 0.363 (LV-PC).

### 3.3. Tensile Strength Obtained from Universal Tensile Tests

**Figure 2:**
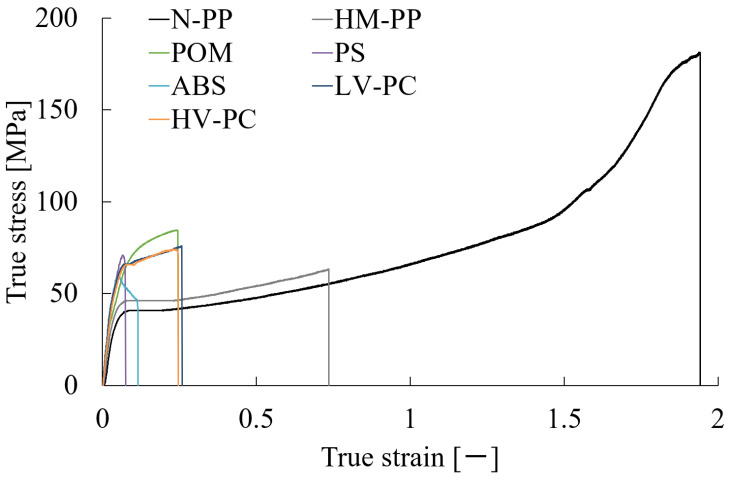
True stress–true strain curves for various polymeric materials.

To evaluate the tensile deformation behavior and fracture characteristics of various polymer materials by comparison, uniaxial tensile tests were performed. Then the true stress–true strain relations were calculated from the obtained stress–strain curves. Figure 2 presents a comparison of the true stress–true strain curves for representative materials.

In the true stress–true strain curves depicted in Figure 2, N-PP exhibited the highest deformability among all samples. In fact, N-PP elongated smoothly without fracturing until reaching a true strain of approximately 1.9. Prominent strain hardening was observed, particularly in the later stage of deformation (at a true strain of 1.0 and beyond). The final true stress at break reached approximately 185 MPa, suggesting that reinforcement caused by molecular chain orientation is dominant. By contrast, HM-PP (high-rigidity polypropylene), which belongs to the same polypropylene family, underwent early fracture at a true strain of approximately 0.7. The marked reduction in ductility compared to N-PP is attributed to the suppression of molecular chain slipping and reorientation, which are caused by differences in crystallinity or by additives associated with increasing rigidity.

Neither PS nor ABS showed any distinct yielding behavior, but both fractured with a brittle character in the extremely low-strain region of less than 0.1 true strain. This finding confirms that these materials are in a glassy state at room temperature and that they have a low capacity to absorb impact energy via plastic deformation. POM, a crystalline polymer, recorded the highest yield stress (approximately 85 MPa) among the measured targets. However, its steady elongation region after yielding was short, leading to fracture at a true strain of approximately 0.3, which indicates that whereas POM possesses high rigidity, its toughness is limited during large deformations. The amorphous PC group (LV-PC and HV-PC) followed a typical ductile fracture process. After reaching the yield point, distinct necking (localized contraction) and accompanying strain softening were observed. Subsequently, through a steady elongation region where deformation progressed under a nearly constant stress, final fracture occurred at a true strain of approximately 0.25.

Table 5 presents evaluation results of tensile modulus (TM), Poisson’s ratio in tension (υ_t_), and maximum true stress (TS) determined by the tensile tests. As shown in Table 5, PS, an amorphous polymer, exhibited the highest TM value (2193 MPa), followed by ABS (2007 MPa) and LV-PC (2014 MPa) in descending order. The TM of N-PP showed the lowest value at 1356 MPa. Results confirmed that the TM of HM-PP (1626 MPa), a similar polypropylene material, was higher than that of N-PP. Regarding υ_t_, N-PP showed the minimum value (0.261) and HV-PC showed the maximum value (0.383). Overall, a tendency for PC-based materials (LV-PC, HV-PC) to exhibit high υ_t_ values was observed. Regarding the TS, N-PP showed a remarkably high value of 184.0 MPa, whereas the other polymer groups remained in the range of 60.0 MPa to 84.6 MPa. Particularly, the TS of HM-PP was 61.9 MPa, which was markedly lower than that of N-PP.

The fact that amorphous polymers with rigid molecular chain backbones, such as PS and ABS, exhibit high TM is considered to be attributable to the low mobility of amorphous chains in the glassy state. Regarding the comparison between N-PP and HM-PP, the phenomenon whereby TM increases while TS decreases considerably suggests that changes in the crystal structure and differences in the localized orientation state of the molecular chains associated with high elastic modulus induce embrittlement of the entire material. Differences in υ_t_ are thought to reflect variations in the free volume and molecular chain network deformation behavior of each polymer. Particularly, the high υ_t_ values exhibited by PC-based materials are consistent with their characteristics of being prone to changing shape under tensile stress without volumetric changes, i.e., possessing excellent ductility. These findings strongly support the hypothesis related to the effects of primary and higher-order structures of polymers on their macroscopic mechanical properties.

### 3.4. Analytical Derivation of Critical Expansion Stress (CES)

To evaluate the multiaxial failure thresholds of the tested thermoplastics, the Critical Expansion Stress (CES) was derived analytically by integrating the macroscopic DuPont impact energy with microscopic shear properties. Based on the energy balance framework, the volumetric strain energy absorbed up to fracture, U, is expressed in the integral form as presented below [15].

(16) $$U=\int \frac{4}{3}G\left(\frac{1-f_{0}}{f_{0}}\right)\varepsilon_{v}d\varepsilon_{v}$$

In that equation, G is the shear modulus obtained from the short-beam shear tests. Also, ε_v_ represents the expansion strain associated with void generation. The critical void volume fraction, f_0_, which triggers unstable nanovoid growth, is identified theoretically by Equation (17).

(17) $$f_{0}=\left(\frac{1}{1+\frac{G}{2\sqrt{3}\tau_{y}}}\right)$$

By applying the experimentally obtained values of E_50_ (Table 3), G, and τ_y_ (Table 4) to this mathematical model, the intrinsic microstructural parameters (f_0_ and CES) were quantified for each polymer grade. The calculated results and their corresponding standard deviations are presented as Table 6.

Compared to N-PP, a crystalline commodity polymer, HM-PP with enhanced rigidity showed lower f_0_ of 0.164, but its CES was higher: 27.1 MPa. In addition, POM, a highly crystalline engineering plastic, exhibited a CES of 28.3 MPa, which was the highest among all the materials, but its standard deviation (S.D.) showed wide scattering of 13.0 MPa. In contrast to these crystalline polymers, PS, an amorphous brittle polymer, remained at low values for both f_0_ (0.139) and CES (15.7 MPa). However, ABS, a rubber-toughened amorphous polymer, exhibited the minimum CES value of 13.9 MPa, yet maintained a high f_0_ value of 0.189. Furthermore, in polycarbonate (PC), an amorphous ductile polymer, a trend was confirmed whereby f_0_ increased from 0.159 to 0.191 and CES increased from 16.7 MPa to 22.0 MPa as the molecular weight increased from the low-viscosity grade (LV-PC) to the high-viscosity grade (HV-PC).

## 4. Discussion

### 4.1. Polymer Physics and Local Relaxation Mechanisms Governing CES

The analytical results presented in Section 3.4 (Table 6) demonstrate that the CES can be derived by coupling the dynamic fracture energy (E_50_) with quasi-static shear parameters (G and f_0_). From the perspective of polymer physics and viscoelasticity theory, equivalently linking these distinct deformation rates requires rigorous cross-examination of local relaxation mechanisms.

Thermoplastic resins typically exhibit pronounced strain-rate dependence. Under high-speed dynamic loading, segmental motions cannot keep pace with the deformation rate, macroscopically shifting the polymer behavior toward a more rigid or glassy regime. However, at the microscopic site of out-of-plane impact directly beneath the indenter, a severe stress concentration field alters the local physics. In this highly localized zone, the timescales of stress-induced free volume expansion, local crystalline lamellae slipping, and local adiabatic heating become highly dominant over the bulk elastic response.

In fact, the phenomenon by which large deformation induced by multiaxial stress concentration dramatically activates the molecular mobility of glassy polymers (promoting substantial molecular relaxation) has been demonstrated by Lee et al. Furthermore, the process by which extremely localized adiabatic heating under high-speed impact deformation causes thermal softening of the material is strongly supported by the high-speed dynamic mechanical analysis of Sarva et al. [23,24]: even under high-speed impact, a marked degree of molecular relaxation is promoted in the extremely localized region serving as the initiation point of fracture because of thermal and mechanical softening. As a result, its local response can be approximated as an extension of the mechanical parameters under quasi-static deformation. Furthermore, regarding the formulation of the CES in this model, the effect of the local increase in matrix rigidity (increase in dynamic modulus) caused by dynamic deformation is canceled out by the dynamic change in the impact fracture energy E_50_ (increase in load response) caused by their numerator–denominator relation. This linkage (scaling effect) between yield/rigidity characteristics and macroscopic dynamic energy response across widely various strain rates finds strong theoretical support in the analysis of tensile yield behavior over a wide rate range reported by Bauwens-Crowet et al., as well as the formulation of polymer plastic flow laws covering up to high velocity ranges by Major and Lang [25,26]. These pioneering studies mechanically rationalize the plausibility of the great degree of cancellation of rate dependence (preservation of correlation) which occurs in the ratio calculation process. Here, it is crucially important to define the boundary conditions of this cancellation hypothesis. This assumption applies primarily to the initial plastic deformation resistance of the matrix before any extreme microstructural rearrangement occurs. For highly ductile polymers that exhibit significant strain hardening or molecular orientation during quasi-static tests, using the final fracture properties directly might disrupt this numerical balance. Therefore, a refined formulation reflecting the stress state before massive strain hardening is necessary for such specific materials, as discussed in detail in Section 4.3. However, an important prerequisite for this scaling hypothesis to hold universally across diverse structural polymers is that the compared macroscopic dynamic parameter (E_50_) and the quasi-static baseline (TS) must reflect corresponding states of mechanical deformation. For highly ductile polymers capable of undergoing extensive cold-drawing and subsequent strain hardening (such as N-PP), the standard quasi-static TS represents an ultimate stress state highly reinforced by mature molecular chain orientation. By contrast, under high-speed multiaxial impact, the material often reaches its critical expansion threshold (CES) and fails prematurely before such large-scale structural rearrangement can manifest. Therefore, the cancellation of rate dependence remains valid only when the baseline reference stress is evaluated at the equivalent deformation stage: specifically, the onset of plastic flow before strain hardening. Acknowledging this boundary condition does not undermine the universality of the proposed model. Rather, it refines the operational definition of the reference matrix resistance, allowing the scaling law to encompass both brittle and highly ductile thermoplastics consistently. However, it must be acknowledged that this cancellation hypothesis involves a degree of oversimplification. As highlighted in previous studies, nonlinear viscoelastic behavior in polymers can significantly modify the material response under high strain rates, which introduces scientific risks when modeling rate dependence solely through quasi-static parameters. While our results suggest that the local molecular relaxation effectively captures the baseline threshold, this approach should be viewed as a phenomenological scaling law rather than a rigorous viscoelastic proof.

Currently, the assumption that rate-dependent changes in dynamic energy response and matrix rigidity cancel each other remains a phenomenological scaling hypothesis or a practical engineering approximation. While direct high-speed mechanical characterization (e.g., high-rate DMA or tensile testing) was not performed in this study, the robust correlation observed between the derived CES and the quasi-static tensile strength suggests that this scaling captures the baseline material thresholds effectively. Future work involving instrumented high-speed testing will be essential to rigorously substantiate the invariance of this multi-axial viscoelastic response.

From an engineering perspective, this rate-dependence cancellation operates as a practical approximation for predicting multiaxial impact failure thresholds. While polymers possess structurally distinct viscoelastic features, the proposed framework intentionally bypasses complex, high-rate experimental characterizations by utilizing universally accessible quasi-static baselines. This methodology does not assume an absolute dynamic invariance across all strain rates; rather, it establishes a phenomenological scaling law where the initial plastic flow resistance effectively captures the baseline material-specific expansion resistance. Consequently, the reliance on straightforward quasi-static parameters, balanced with the physics of localized molecular relaxation, enhances the practical robustness of this model, making it an versatile tool for high-fidelity CAE calibration without the need for sophisticated dynamic testing equipment.

### 4.2. Materials Dependence of Microscopic Damage and Fracture Mechanisms

The material-dependent variations in f_0_ and CES presented in Table 6 reflect the distinct physical constraints imposed by the respective polymer matrix structures and their inherent deformation modes.

For the crystalline polymers (HM-PP and POM), the high CES values (27.1 MPa and 28.3 MPa, respectively) are deeply linked to the highly developed crystalline lamellar phase, which acts as a robust mechanical barrier against volumetric expansion of the matrix [27]. However, the fact that the standard deviation is remarkably large in POM is thought to arise from instability of the brittle void coalescence process characteristic of highly crystalline matrices. This instability of void coalescence and the accompanying sudden fracture propagation, unique to crystalline polymers, is a characteristic that is consistent with an earlier report by Pawlak et al., who systematized the void coalescence process under high constraint [28].

By contrast, the amorphous brittle PS matrix exhibits markedly lower CES (15.7 MPa) and f_0_ (0.139). The behavior of PS, where both f0 and CES are low, clearly reflects its brittle fracture characteristics, in which crazes easily initiate and grow under low stress, reaching the critical void volume fraction without involving plastic deformation of the entire matrix. Such craze-preferential fracture behavior under low stress in glassy polymers shows extremely high consistency with Kramer’s deformation mechanism theory [29], which holds that microscopic void growth inside crazes leads to sudden fracture. In contrast, the reason why ABS achieves both a minimal CES and a large f0 is suggested to be that the rubber particles dispersed in the matrix preferentially undergo cavitation under low expansion stress, but the subsequent rapid expansion of voids or propagation into cracks is suppressed effectively by shear yielding of the surrounding amorphous matrix. This cooperative effect of early cavitation of rubber particles and the matrix shear yielding induced by it is also mechanically supported by the toughening mechanism theories of Bucknall, who formulated the fracture mechanism of rubber-modified plastics, and by work performed by Borggreve et al., who reported the role of the matrix in the transition of deformation modes [30,31]. Regarding the molecular weight dependence in PC, it can be inferred that the increase in the entanglement density of polymer chains (HV-PC) suppresses both void expansion (increase in CES) and the local rupture and coalescence of the matrix film separating the voids (increase in f_0_). In fact, Pitman et al. reported from an elasto-plastic model that the craze stress and strain energy release rate increase with the increasing molecular weight of PC. This pattern of increase strongly supports the mechanical interpretation from this study that the increase in entanglement density simultaneously controls void expansion (CES) and matrix film rupture (f_0_) [32].

From the calculation results and discussion presented above, it was confirmed that the model of critical expansion stress using the DuPont impact failure energy density proposed herein captures the qualitative trends shown by these materials.

### 4.3. Comparison of “Critical Expansion Stress” Among Materials and Identification of Impact-Resistance Bottlenecks

**Figure 3:**
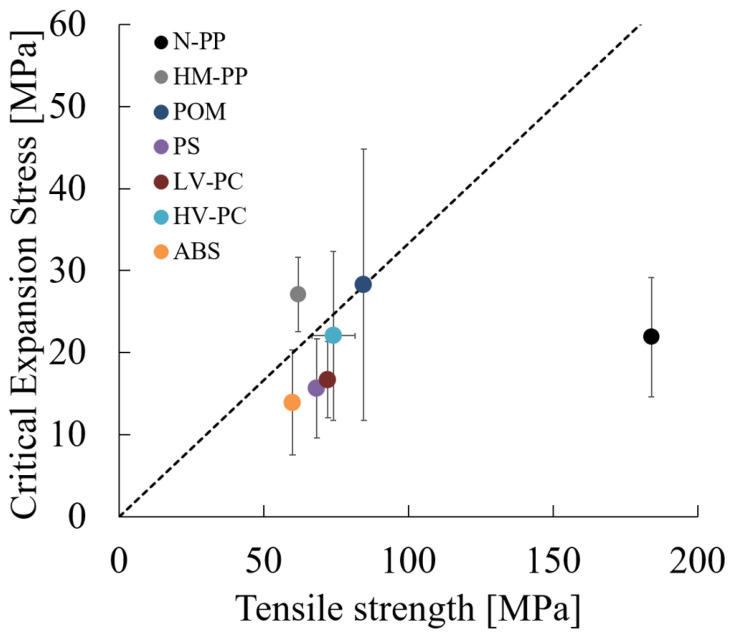
Correlation between tensile strength (TS) and critical expansion stress (CES) in various polymer materials. The dashed line passing through the origin represents the linear baseline correlation between the critical expansion stress and tensile strength according to Equation (18).

This section presents verification of the quantitative validity of the method proposed herein by comparing the maximum true stress results obtained in Section 3.3 with the critical expansion stress (CES) determined in Section 3.4. To clarify the correlation between tensile strength and critical expansion stress (CES) in various polymers, their measured values were shown and compared. Figure 3 presents the relation between the critical expansion stress and the maximum true stress for each material. In Figure 3, the dotted line represents the point at which the CES is 1/3 of the tensile strength (TS). This dotted line was established because the hydrostatic stress generated by a uniaxial tensile load is given by the following Equation (18).

(18) $$CES=\frac{TS}{3}$$

From a strict macroscopic continuum mechanics perspective, the theoretical hydrostatic stress component under an equi-biaxial tensile field with a thickness constraint (such as the DuPont impact test) is inherently higher than that under a uniaxial tensile state. Therefore, a direct equivalence between the derived CES and TS/3 requires a micro-mechanical bridging logic.

In a real polymer matrix, microscopic heterogeneities (e.g., crystalline lamellar boundaries or rubber particle interfaces) govern the expansion fracture process. Once a nanovoid nucleates under multiaxial stress, the severe macroscopic triaxial constraint is locally relaxed. Then the micro-matrix immediately surrounding the void facet undergoes highly directional, peel-like stretching rather than retaining an ideal triaxial constraint field. Furthermore, the non-spherical, prolate spheroidal growth of voids along the principal stress direction reshapes the local stress triaxiality, thereby mitigating the high macroscopic constraint.

Consequently, the empirical adherence of the experimentally obtained data to the “1:3 rule” across multiple polymers does not imply that the macroscopic stress field during the DuPont test is identical to uniaxial tension. Rather, it demonstrates that the intrinsic dynamic threshold triggering unstable volume expansion is uniquely scaled by the hydrostatic stress level (TS/3) that the matrix polymer can physically sustain immediately before moving into the large-scale plastic flow or strain-hardening regime under quasi-static conditions. In other words, the quasi-static uniaxial parameters act as an accessible baseline reference scale for the material-specific expansion resistance, thereby bridging the macro-micro stress state discrepancy.

In other words, if each data point is distributed near this dotted line, the maximum true stress obtained during the tensile test can be explained theoretically by the critical expansion stress determined by the DuPont impact test. For the HM-PP, POM, PS, LV-PC, HV-PC, and ABS samples, the data points were generally distributed near this dotted line. A trend was observed by which the CES increased in proportion to the tensile strength. It is noteworthy that, whereas the tensile strength of N-PP showed an extremely high value of approximately 145 MPa compared to the other materials, its CES remained at about 22 MPa, confirming a behavior that deviates markedly downward from the proportional relation indicated by the dotted line. This behavior will be discussed by comparing the fracture morphology after the DuPont impact test with that after the uniaxial tensile test.

**Figure 4:**
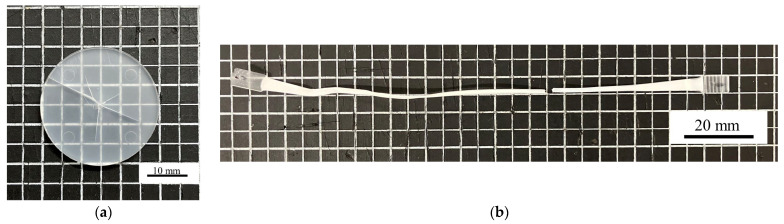
Morphological observations of fractured N-PP specimens after mechanical testing: (**a**) Optical micrograph of a disc-shaped specimen subjected to drop-weight impact testing, illustrating radial crack propagation from the center point of impact. (**b**) Optical micrograph of a tensile specimen fractured under uniaxial loading, showing distinct necking behavior and localized plastic deformation before complete failure.

Figure 4 shows the N-PP fracture morphologies after the DuPont impact test and after the uniaxial tensile test. Multiple cracks radiating from the impact point were observed in the disc-shaped specimen subjected to the DuPont impact test (Figure 4a). At this time, macroscopic ductile deformation and whitening phenomena around the cracks were extremely limited, indicating that the material fractured in a brittle manner, without local plastic deformation. However, in the specimen after the uniaxial tensile test, the occurrence of a great degree of necking and its subsequent propagation throughout the parallel gauge section were confirmed before reaching fracture (Figure 4b). The final fracture occurred in a sufficiently stretched and oriented (strain-hardened) region, exhibiting an extremely large elongation at break, in contrast to that observed during the impact test.

These differences in fracture morphology are regarded as attributable to the deformation-rate-dependent relaxation behavior of polymer chains and the resulting degree of achievement of strain hardening. Under low-speed deformation such as that of a uniaxial tensile test, sufficient time exists for the orientation and rearrangement of polymer chains to keep pace with the external force. Consequently, stable plastic deformation accompanied by necking progresses, leading to fracture in the strain-hardening region. By contrast, under high-speed deformation such as the DuPont impact test, the molecular motion cannot keep pace with the deformation rate. Moreover, strain hardening is not sufficient to disperse or relax local stress concentration. Consequently, it can be inferred that a brittle fracture point is selected before strain hardening occurs. In turn, this interpretation suggests that the fracture process of N-PP is governed by the timing of the onset of strain hardening depending on the applied deformation rate. This competition between the timescales of the deformation rate and strain hardening shows extremely high consistency with the theoretical model of Schrauwen et al., which states that strain hardening suppresses local fracture in the intrinsic deformation of crystalline polymers, and the crystal plasticity theory of Pawlak and Galeski, which states that the structural rearrangement of polymer chains according to the loading rate governs the deformation mode [28,33].

**Figure 5:**
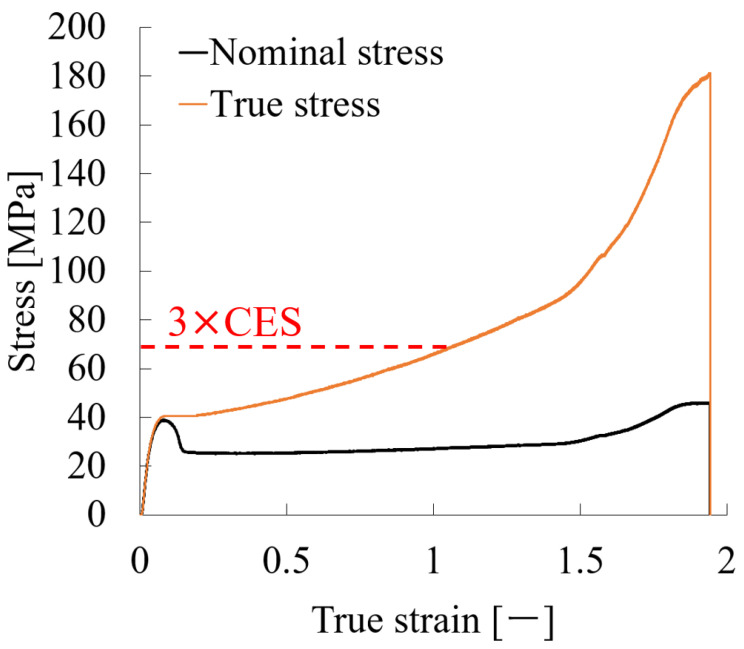
True stress–true strain curve of N-PP. The “3 × CES” in the figure represents three times the critical expansion stress.

Based on the considerations presented above, we confirmed which region of the true stress–true strain curve of N-PP corresponds to three times the critical expansion stress. Figure 5 shows the true stress–true strain curve of N-PP. The stress level of three times the critical expansion stress (3 × CES, approximately 68 MPa), indicated by the red dashed line in the figure, intersected the true stress–true strain curve at a true strain of approximately 1.3, immediately before the onset of strain hardening. This intersection suggests that, under high-speed deformation such as the DuPont impact test described in the discussion presented above, a brittle fracture point is selected before strain hardening occurs.

**Figure 6:**
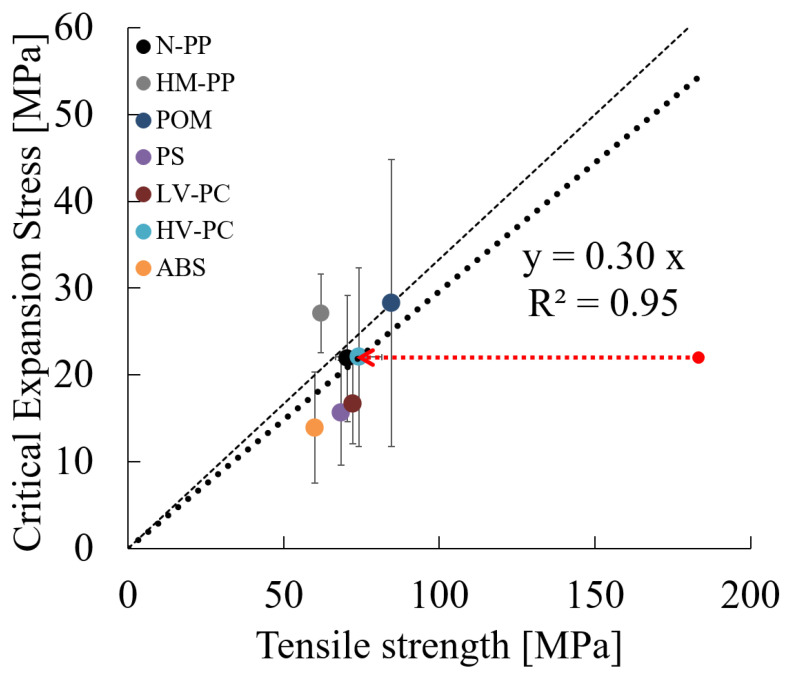
Correlation between critical expansion stress and tensile strength for various polymeric materials, including N-PP, HM-PP, POM, PS, LV-PC, HV-PC, and ABS. The dotted line represents the linear regression line (CES = 0.30 TS, R^2^ = 0.95). The dashed line passing through the origin represents the linear baseline correlation between the critical expansion stress and tensile strength according to Equation (18). The dotted red arrows indicate the changes in these mechanical parameters before and after the modification process.

Based on this inference, the maximum true stress of N-PP depicted in Figure 3 was corrected to the true stress immediately before strain hardening occurs. The corrected results are shown in Figure 6. Data incorporating the initial tensile strength of N-PP (approximately 184 MPa) (red symbol in the figure) deviated markedly to the right from this common trend. However, based on the logic presented above, as a result of re-plotting by re-defining the true stress value immediately before the onset of strain hardening as the tensile strength (black symbol in the figure), the data for N-PP agreed extremely well with the correlation shown by the other polymer materials. These results suggest that the critical expansion stress in polymer materials depends more strongly on the stress level in the early stage of plastic deformation before strain hardening becomes manifested, than on the final fracture strength of the material. This material-dependent modification does not contradict the claimed universality of our model; rather, it underscores it. Although the dynamic structural mismatch in N-PP causes an apparent deviation from the baseline linear relation, redefining the representative tensile strength based on the pre-hardening flow state restores the universal 1:3 correlation. Consequently, the proposed CES framework captures the intrinsic hydrostatic threshold triggering multiaxial cavitation across diverse polymer species, provided that the appropriate matrix deformation stage is selected as the baseline. The fact that a consistent proportional relation holds between the CES and tensile strength in many polymers implies that the fracture mechanism involving expansion is essentially governed by the deformation resistance of the matrix before being affected by strengthening because of the high orientation of polymer chains. In particular, the fact that a universal relation with other materials was obtained for N-PP using the stress before strain hardening indicates that when evaluating material strength under multiaxial stress or high-speed deformation, the stress state before strain hardening can be a more appropriate physical indicator than the nominal fracture strength obtained from static tests.

### 4.4. Practicality of the Proposed Method and Future Prospects (And Limitations) for Extension to CAE Analysis

This section presents verification of the validity of the estimation method for critical expansion stress based on the DuPont impact test results, established up to the preceding section, along with discussion of its engineering practicality and future limitations. As depicted in Figure 6, a comparison between the critical expansion stress calculated from the drop-weight impact test and the true-stress-based tensile strength obtained from the tensile test confirmed a trend whereby the ratio between the two generally matches 1/3. In a theoretical state of uniform uniaxial tensile stress, the triaxiality of stress is strictly 1/3, irrespective of material properties (such as molecular orientation or dispersed particles). For this study, to avoid confusion with the general stress triaxiality in continuum mechanics (the ratio of hydrostatic stress σ_m_ to equivalent stress $\bar{\sigma }$), the ratio of the critical extension stress CES to the experimentally obtained uniaxial tensile strength TS derived from the DuPont impact test was defined as “apparent stress triaxiality χ_app_” as shown in Equation (19) below. It is used as an indicator of the fracture behavior of each material.

(19) $$\chi_{app}=\frac{CES}{TS}$$

**Figure 7:**
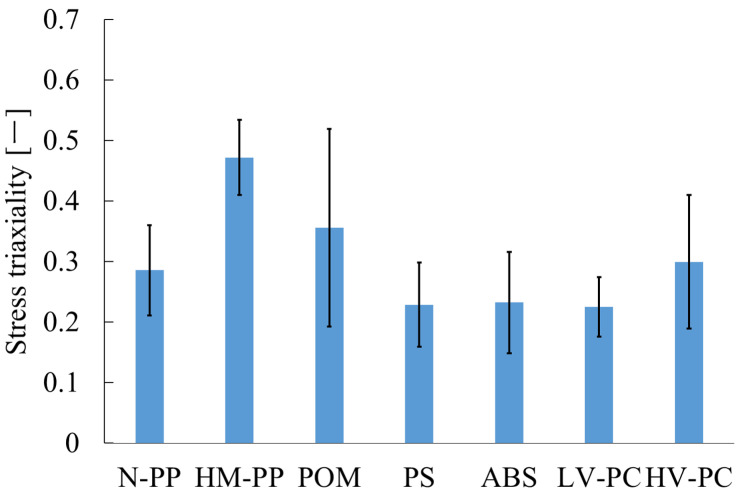
Comparison of stress triaxiality among various polymer materials. The bar graph presents the mean values of the stress triaxiality calculated for the polymer species. The error bars represent the standard deviations of the measurements.

This definition ensures that for an ideal isotropic material under uniaxial tension, χ_app_ theoretically converges to 1/3, consistent with the ‘1:3 rule’ (σ_m_/σ = 1/3) used throughout this study. By aligning the text with Equation (19), we clarify that CES represents the hydrostatic component triggering expansion failure relative to the matrix’s tensile resistance. Figure 7 shows the apparent stress triaxiality of each material obtained from this study. As the figure shows, clear material-type-dependent differences are apparent in the mean values and scattering of the stress triaxiality. Among the polymer materials examined, HM-PP exhibited the highest stress triaxiality (approximately 0.47). By contrast, the stress triaxiality values of PS, ABS, and LV-PC remained low values at approximately 0.22 to 0.23. No significant difference was found among these materials. Although the mean value for POM showed a high value of approximately 0.35, the range of the error bars was extremely wide compared to the other materials. Scattering of the measured values was remarkable. Furthermore, regarding comparison of the effects of viscosity in polycarbonate (PC), the stress triaxiality of the high-viscosity product, HV-PC (approximately 0.30), showed a higher value than that of the low-viscosity product, LV-PC (approximately 0.22). What is noteworthy here is that for PS, ABS, LV-PC, and HV-PC, data points were confirmed where the stress triaxiality fell below 1/3, which is the theoretical value in a general uniform uniaxial tensile state.

In an ideal uniaxial tensile state of an isotropic material based on continuum mechanics, the stress triaxiality theoretically converges to 1/3. However, in the actual microscopic deformation process of real resins, slight deviations from this theoretical ratio can occur because of complex factors of polymer physics. First and foremost is the effect of the dynamic change in Poisson’s ratio accompanying plastic deformation and plastic anisotropy. Particularly in injection-molded products, the polymer chains are oriented in specific directions because of flow orientation during molding, which induces directional dependence (plastic anisotropy) in the shear yield characteristics under multiaxial stress loading. Consequently, the development of effective stress shifts from that of an ideal isotropic material. Second, the non-spherical growth (anisotropic expansion) of voids can be cited. Unlike the spherical voids assumed in models such as the Gurson model, actual voids originating between crystalline lamellae or at rubber particle interfaces stretch anisotropically (deform into a prolate spheroidal shape) along the principal stress direction. This geometric asymmetry redistributes the local stress triaxiality field around the voids, acting as a factor that decreases or increases the macroscopic expansion stress response. Furthermore, regarding thermal effects under high-speed deformation, extremely localized adiabatic heating can potentially induce minor thermal softening at the very final stage of void coalescence, but its contribution to the baseline deviation of the apparent stress triaxiality is considered secondary. If thermal softening were the primary governing factor, then it would conflict with the core assumption verified in Section 4.1: that rate-dependent viscoelastic effects are greatly canceled out. Therefore, the observed baseline deviation from the theoretical 1/3 ratio should be primarily attributed to the previously described mechanical and structural factors such as molding-induced plastic anisotropy and geometrical void asymmetry, rather than to dominant thermal adjustments. Nevertheless, a more rigorous decoupling of these coupled mechanical–thermal effects remain as a task for future high-rate validation by experimentation.

Taken together, these results suggest that the molecular backbone, aggregation state, and molecular weight of polymers strongly influence the degree of plastic constraint during deformation. The fact that high stress triaxiality exceeding 1/3 was obtained in HM-PP reflects a strong plastic constraint because of high matrix rigidity and crystal structure. It is thought to act as a factor promoting development of a three-dimensional hydrostatic stress state inside the material. Consequently, fracture is considered to have occurred in the necking propagation region in the tensile test.

For PS, ABS, and LV-PC, with stress triaxiality less than the reference value of 1/3 for uniaxial tension, the local plastic constraint was relaxed under tensile loading, indicating a stress state in which shear plastic deformation (such as the formation of shear bands) is predominant inside the material. Generally, a decrease in stress triaxiality engenders a relative decrease in hydrostatic tensile stress, which is the primary factor driving the initiation and growth of voids. Therefore, in the group of materials falling below 1/3, the formation of crazes depending on hydrostatic stress and the brittle void elongation process are suppressed. Instead, ductile shear deformation behavior accompanied by the relaxation of stress concentration predominantly progresses. Because the maximum true stress became more than three times the CES, the stress triaxiality became smaller than the theoretical value. This mechanism varies depending on the material. For PS and LV-PC, the factor is regarded as the manifestation of mechanical anisotropy caused by the strong orientation of molecules in the parallel gauge section of the dumbbell specimen. For ABS, it is considered that the butadiene rubber particles dispersed in the molded product act as stress concentration sources. Numerous local shear deformations (or fine void formations) progress at the interface with the matrix, thereby efficiently relaxing the plastic constraint of the entire material. Furthermore, the behavior in PC where the stress triaxiality increases concomitantly with increasing viscosity (molecular weight) is consistent with the general principle of materials science that an increase in the entanglement density of polymer chains suppresses local shear deformation (formation of shear bands), consequently increasing the plastic constraint pressure inside the material.

The wide scattering of data observed for POM is inferred to be attributable to the fact that the non-uniform spherulitic structure and local differences in orientation, characteristic of highly crystalline polymers, alter the degree of stress concentration during deformation.

What is particularly noteworthy as a conclusion of this study is that the CES calculated using mechanical parameters obtained from such quasi-static tests (short-beam shear test and uniaxial tensile test) can rationally explain the DuPont impact test results, which is a high-speed deformation process. Inherently, polymer materials exhibit significant viscoelasticity (time-temperature superposition principle), and because the elastic modulus and yield stress increase with an increasing deformation rate, a concern arises that dynamic parameters should be applied to evaluate behavior during impact. However, in the process of CES derivation in the proposed model, both the local increase in material rigidity caused by high-speed deformation (increase in dynamic shear modulus) and the accompanying dynamic change in impact fracture energy mutually contribute to the theoretical equation (the ratio calculation process). As a result, it is highly probable that the rate-dependence effect is offset to a great degree (cancelled). In other words, the calculated CES can be interpreted as strongly reflecting the characteristics of an “intrinsic critical threshold of the material,” which are governed by the microscopic structure (the constraining force of the crystalline phase and the entanglement density of polymer chains) when the material undergoes volumetric expansion (cavitation), rather than by the timescale of the deformation rate itself. Therefore, the fracture threshold during high-speed out-of-plane impact can be uniquely predicted solely by a combination of versatile quasi-static mechanical tests, bypassing complex dynamic viscoelastic evaluation. That fact underscores the engineering practicality and validity of the proposed method.

Compared to the criteria established by Bucknall et al. [16] and Altenbach et al. [17], which often demand complex notch geometries or ultra-high-pressure testing environments, the CES approach offers a distinct measurable advantage: it identifies the multiaxial fracture threshold uniquely from only two physically clear variables—the intrinsic shear modulus (G) and the initial porosity (f_0_). By utilizing existing, simple testing equipment (DuPont impact, short-beam shear, and tensile testers) without the need for sophisticated dynamic or multiaxial setups, this methodology enables a cost-effective and time-efficient screening of a material’s brittle–ductile transition. This simplicity facilitates the direct integration of these material constants into CAE failure sub-components, providing a practical ‘first-order screening tool’ for high-fidelity impact simulations.

Furthermore, this law held true for both crystalline and amorphous polymers. That fact suggests an extremely limited influence of differences in microscopic fracture mechanisms, such as cavitation and craze initiation, on the applicability of this law [16,17]. The engineering and practical significance of this finding is noteworthy. Conventional DuPont impact tests have been limited to qualitative pass/fail judgements of products or the calculation of macroscopic fracture potential energy depending on the specimen thickness and equipment geometry. However, using this method, it becomes possible to extract quantitative material properties intrinsic to the material while using the existing simple testing equipment as it is. This extraction makes it possible to acquire highly versatile quantitative indicators in the product design process while suppressing experiment-related costs and time.

Furthermore, the extracted critical expansion stress is expected to be deployed as a failure criterion in computer-aided engineering (CAE) analysis, particularly in the finite element method. When an impact load under a multiaxial stress state is applied to plastic products, it has been difficult to reproduce the fracture behavior accurately using conventional predictions based on uniaxial tensile yield stress [34]. By incorporating the critical expansion stress obtained using this method into simulations as a critical parameter for fracture initiation in a multiaxial stress field, the prediction accuracy of out-of-plane impact properties will be improved dramatically, enabling more sophisticated structural design.

Several limitations might constrain the application of this proposed method. For this verification, the critical expansion stress obtained from a dynamic (high-strain-rate) event, specifically the drop-weight impact test, is compared with the maximum true stress obtained from a static (low-strain-rate) tensile test. The assumption that the rate-dependent sensitivity of the matrix dynamic shear modulus (G) and the macroscopic fracture energy (E_50_) match systematically across structurally distinct polymers (crystalline vs. amorphous, rubber-modified vs. glassy) remains a strong simplification. Because direct high-speed constitutive relations and dynamic viscoelastic properties were not measured using high-rate testing instruments for this study, assuming a perfect and universal cancellation of rate dependence across all selected resins introduces a logical limitation. Consequently, this framework should be evaluated as an operational ‘engineering approximation’ and a first-order screening tool rather than a final material law for direct CAE embedding. While it bypasses complex dynamic characterization, it provides a robust baseline for material selection and initial structural design, which can be further refined through high-speed validation and consideration of specimen anisotropy. Moreover, the effects of material anisotropy (orientation) occurring in actual injection-molded products and the scale effect associated with variations in specimen thickness are omitted from this model [34,35]. Therefore, although the proposed method possesses sufficient practicality and extensibility to CAE as a quantitative evaluation guideline for the out-of-plane impact properties of plastic materials, a direct comparison with high-speed tensile test data under identical dynamic strain rates is an indispensable next step to validate the underlying physics of this fracture mechanism rigorously and to improve prediction accuracy. A critical limitation of the current framework is the absence of direct high-speed mechanical characterization, such as high-rate tensile or dynamic mechanical analysis (DMA). Consequently, the proposed model currently sits between a robust physical theory and a practical engineering approximation. To provide a more solid foundation, future work should involve temperature sensitivity and model parameter-sensitivity analysis to further substantiate the invariance of this multi-axial viscoelastic response under extreme conditions. At present, this methodology is best positioned as a first-order screening tool for high-fidelity CAE calibration.

To enhance the predictive accuracy and physical validity of the CES model, subsequent research will focus on direct high-velocity validation and temperature-dependent characterization. Integrating these dynamic factors will be an indispensable step toward embedding this model as a rigorous material law into commercial CAE platforms. These challenges are expected to serve as important steps toward expanding the scope of application of this method further and toward deepening the fracture mechanics of polymer materials.

## 5. Conclusions

For this study, a new mechanical model based on the deformation and fracture behavior of the DuPont impact test was constructed, aiming at the quantification of failure criteria under multiaxial stress for thermoplastic materials. Then a method for evaluating the “Critical Expansion Stress (CES)” was proposed. The primary findings obtained from this study are presented below.

The DuPont impact test using the staircase method was conducted on five types and seven grades of materials, including PP, POM, PS, ABS, and PC, for rigorous calculation of the out-of-plane impact absorbed energy per unit volume considering the scale effect.

Based on the shear mechanical properties of the materials obtained from the short-beam shear test, the microscopic parameters (the shear modulus G and the critical void volume fraction f_0_) were theoretically identified. By integrating these into a mathematical model, we derived the critical expansion stress, which is an intrinsic threshold for each material.

The derived CES followed a universal ‘1:3 rule’ relative to the tensile strength across diverse polymer species. This confirms that the proposed framework can uniquely transform conventional qualitative DuPont results into quantitative physical constants, offering a new mechanics-of-materials platform for material screening and CAE calibration.

The results demonstrate that the proposed critical expansion stress serves as a practical screening indicator for evaluating the out-of-plane impact properties of thermoplastics within the scope of the proposed engineering approximation. Future work will expand the scope of this method to include diverse molding conditions and specialized materials, such as fiber-reinforced polymers. To move beyond a first-order screening tool, subsequent research will incorporate direct high-velocity tensile testing, temperature-sensitivity analysis, and DMA to rigorously validate the underlying viscoelastic physics. Furthermore, we aim to implement this CES model into finite element analysis (FEA) platforms, performing model parameter-sensitivity studies to achieve high-precision fracture simulations in complex industrial product geometries.

## Figures and Tables

**Table 1:** Specifications and melt flow rate (MFR) measurement conditions of the thermoplastic polymer materials used for this study.

Material	Code	Grade	MFR
Polypropylene	N-PP	Novatec PP MA1B	21 g/10 min@190 °C, 2.16 kg
High-Modulus Polypropylene	HM-PP	Novatec PP MA3H	10 g/10 min@190 °C, 2.16 kg
Polyoxymethylene	POM	TENAC™3010	9 g/10 min@190 °C, 2.16 kg
Polystyrene	PS	Toyo Styrene GP G210C	10 g/10 min@200 °C, 5.0 kg
Low-Viscosity Polycarbonate	LV-PC	lupilon™H3000	30 g/10 min@300 °C, 1.2 kg
High-Viscosity Polycarbonate	HV-PC	lupilon™S2000	10 g/10 min@300 °C, 1.2 kg
Acrylonitrile Butadiene Styrene	ABS	Kralastic GA101	26 g/10 min@220 °C, 10.0 kg

**Table 2:** Injection molding conditions for the preparation of (a) disk specimens and (b) dumbbell specimens of various polymers. N/A denotes “Not Applicable”.

(a) Disk type							
Disk	N-PP	HM-PP	POM	PS	LV-PC	HV-PC	ABS
Pre-heating Temp. [°C], Time [h]	N/A	N/A	N/A	80, 12	80, 12	80, 12	80, 12
Inj. temp. [°C]	230	230	230	230	280	315	250
Mold temp. [°C]	50	50	80	80	130	100	60
Inj. Speed [mm/s]	30	30	20	10	30	10	10
Inj. time [s]	10	10	10	10	10	10	10
Cooling time [s]	15	15	15	15	15	15	15
Holding press. [MPa]	28	56	56	28	56	56	28
(b) Dumbbell type							
Dumbbell	N-PP	HM-PP	POM	PS	LV-PC	HV-PC	ABS
Pre-heating Temp. [°C], Time [h]	N/A	N/A	N/A	80, 12	80, 12	80, 12	80, 12
Inj. temp. [°C]	230	230	230	230	280	315	250
Mold temp. [°C]	50	50	80	40	100	100	60
Inj. Speed [mm/s]	10	10	20	10	30	30	30
Inj. time [s]	10	10	10	10	10	10	10
Cooling time [s]	15	15	15	15	15	15	15
Holding press. [MPa]	56	56	56	28	21	21	28

**Table 3:** Impact failure energy density of various polymer materials obtained from Dupont impact tests.

Code	E_50_ [MJ/m^3^]	s [MJ/m^3^]
N-PP	0.229	0.025
HM-PP	0.227	0.006
POM	0.203	0.069
PS	0.042	0.006
ABS	0.090	0.019
LV-PC	0.056	0.004
HV-PC	0.172	0.038

**Table 4:** Comparison of mechanical property parameters of various polymer materials derived from analysis of short-beam shear test results. Numbers in parentheses represent the standard deviation.

Code	τ_s_ [MPa]	τ_m_ [MPa]	τ_l_ [MPa]	τ_y_ [MPa]	υ [−]	E [MPa]	G [MPa]
N-PP	4.8 (0.4)	6.9 (0.5)	9.0 (0.7)	12.3 (0.7)	0.324 (0.025)	485 (77)	183 (27)
HM-PP	5.7 (0.5)	7.4 (0.7)	9.6 (1.2)	13.4 (1.3)	0.343 (0.033)	638 (152)	237 (53)
POM	6.0 (0.9)	8.1 (1.1)	10.3 (1.3)	14.3 (1.8)	0.342 (0.019)	728 (215)	271 (78)
PS	6.8 (0.6)	9.2 (0.6)	11.7 (0.6)	16.3 (0.6)	0.342 (0.032)	941 (235)	351 (78)
ABS	5.7 (0.1)	7.0 (0.7)	9.0 (0.5)	12.7 (0.7)	0.353 (0.013)	512 (82)	189 (29)
LV-PC	8.4 (0.4)	10.9 (0.7)	13.3 (0.7)	19.1 (0.7)	0.363 (0.013)	954 (72)	350 (25)
HV-PC	7.5 (0.1)	9.6 (0.4)	12.0 (0.8)	17.1 (0.6)	0.356 (0.027)	678 (80)	250 (26)

**Table 5:** Mechanical properties of various polymeric materials obtained from uniaxial tensile tests.

Code	TM [MPa]	S.D. [MPa]	υ_t_ [−]	S.D. [−]	TS [MPa]	S.D. [MPa]
N-PP	1329	50	0.261	0.002	184.0	2.0
HM-PP	1626	58	0.326	0.056	61.9	2.1
POM	1794	31	0.361	0.017	84.6	0.4
PS	2193	94	0.350	0.028	68.4	1.2
ABS	2007	162	0.345	0.026	60.0	0.4
LV-PC	2014	29	0.322	0.022	72.2	1.9
HV-PC	1750	52	0.383	0.004	74.1	7.4

**Table 6:** Summary of f_0_, critical expansion stress (CES), and standard deviation (S.D.) for various polymer materials.

Code	f_0_ [−]	CES [MPa]	S.D. [MPa]
N-PP	0.189	21.9	5.7
HM-PP	0.164	27.1	3.5
POM	0.155	28.3	13.0
PS	0.139	15.7	4.8
ABS	0.189	13.9	5.0
LV-PC	0.159	16.7	3.7
HV-PC	0.191	22.0	8.1

## Data Availability

The original contributions presented in this study are included in the article. Further inquiries can be directed to the corresponding author.
